# N-terminal pro–B-type natriuretic peptide levels in normotensive and hypertensive dogs with myxomatous mitral valve disease stage B

**DOI:** 10.1186/s13620-023-00233-0

**Published:** 2023-02-08

**Authors:** In Sung Jang, Won Kyoung Yoon, Eun Wha Choi

**Affiliations:** 1 Department of Veterinary Clinical Pathology, College of Veterinary Medicine and Institute of Veterinary Science, Kangwon National University, 1 Kangwondaehak-Gil, Chuncheon, Gangwon-Do 24341 Republic of Korea; 2 Guardian Angel Veterinary Hospital, 552, Gyeongsudae-Ro, Dongan-Gu, Anyang-Si, Gyeonggi-Do 14112 Republic of Korea

**Keywords:** Biomarker, Canine, Myxomatous mitral valve disease (MMVD), N-terminal pro-B-type natriuretic peptide (NT-proBNP), Systemic hypertension

## Abstract

**Background:**

Systemic hypertension affects the heart, and to the best of our knowledge, no study has investigated the effects of N-terminal pro-B-type natriuretic peptide (NT-proBNP) in dogs with myxomatous mitral valve disease (MMVD) stage B and systemic hypertension. This study aimed to investigate the blood level of NT-proBNP and assess the selected echocardiographic variables in dogs with MMVD stage B according to the presence of systemic hypertension or normal blood pressure and in dogs without MMVD.

**Results:**

The study group comprised 37 dogs with stage B MMVD (normotensive group, *n* = 30; systemic hypertension group, *n* = 7) and 13 dogs without MMVD. We evaluated NT-proBNP, blood pressure, complete blood count (CBC), and serum chemistry in all 50 dogs. We performed electrocardiography, radiography, and echocardiography on 44 dogs (37 dogs with MMVD and 7 dogs without MMVD). The NT-proBNP concentrations showed significant intergroup differences (*p* < 0.001). Normotensive dogs with MMVD stage B (median [interquartile range]: 1083.5 [574.8–1912.8] pmol/L) and hypertensive dogs with MMVD stage B (2345.0 [1812.5–2533.0] pmol/L) showed significantly higher NT-proBNP concentrations than dogs without MMVD (504 [430–774] pmol/L, *p* = 0.009 and *p* < 0.001, respectively), and dogs in the systemic hypertension group showed significantly higher NT-proBNP concentrations than those in the normotensive group (*p* = 0.046). Mitral valve regurgitation velocity was significantly higher in dogs in the systemic hypertension group (6.11 [6.07–6.24] m/s) than in those in the normotensive group (5.53 [5.17–5.95] m/s, *p* = 0.006). The left atrial to aortic root ratio (LA/Ao), E-peak velocity, and left ventricular end‐diastolic internal diameter corrected for body weight (LVIDDN) were significantly lower in dogs without MMVD than in dogs with MMVD stage B.

**Conclusions:**

These findings suggest that NT-proBNP concentrations are higher in dogs with MMVD stage B with systemic hypertension than in normotensive dogs with MMVD stage B. Therefore, clinicians should be aware that NT-proBNP could be elevated in the presence of systemic hypertension.

## Background

Myxomatous mitral valve disease (MMVD) is the most common acquired heart disease in older dogs characterized with murmur [1], accounting for over 70% of heart diseases. Progressive valve degeneration associated with aging induces valve regurgitation and the resulting backflow of blood is heard as a heart murmur during heartbeat. Valvular insufficiency occurs when the end of the valve becomes thickened like a nodule because of myxomatous degeneration.

Mitral valve prolapse may occur as an aspect of mitral valve degeneration, in which the mitral valve leaflets are abnormally thickened and the valve is displaced to the left atrium during systole, which may cause valvular insufficiency [2]. Myxomatous mitral valve degeneration occurs mainly in small dogs, and Cavalier King Charles Spaniels develop it earlier than other breeds [3]. The atria and ventricles enlarged to compensate for mitral valve regurgitation, and the pressure in the left atrium increased.

Myxomatous mitral valve disease is divided into stages A, B, C, and D according to the guidelines of the American College of Veterinary Internal Medicine (ACVIM) [4]. Blood pressure is the force between the contraction of the heart and the resistance of the blood vessel. Dogs are classified as having systemic hypertension when the systolic or diastolic blood pressure exceeds 160 mmHg or 95 mmHg, respectively [5].

In the clinical setting, cardiac biomarkers with high sensitivity and specificity can be used to predict or diagnose a heart disease. In clinical practice, troponins I and T have been used as predictors of heart disease, and the N-terminal pro-B-type natriuretic peptide (NT-proBNP) concentration is also considered the most reliable predictor of a heart disease [6, 7]. In humans, BNP and NT-proBNP are reliable biomarkers for predicting heart disease or heart failure, and are used in the emergency evaluation of acute heart failure or dyspnea as well as the cardiovascular effects of hypertension [8–11]. The NT-proBNP concentration can be used as an adjunct in the diagnosis of heart failure [11]. A recent study showed that the higher the MMVD stage in dogs, the higher the NT-proBNP concentration. The concentration of NT-proBNP (median [min–max]) was 543 (16–1558) pmol/L in 10 healthy dogs, 677 (24–1344) pmol/L in 10 dogs with MMVD stage B1, 1553 (531–3010) pmol/L in 10 dogs with MMVD stage B2, and 1963 (424–4086) pmol/L in 8 dogs with MMVD stage C which had been treated with medication and were in a stable condition [12]. Previous studies on humans and cats suggested that NT-proBNP concentrations are elevated in hypertension [11, 13], and can be used effectively in the prognosis of hypertension treatment in cats [13]. In humans, hypertension affects the myocardium and increases NT-proBNP concentration.

To the best of our knowledge, no study has compared NT-proBNP concentrations in normotensive and hypertensive dogs with MMVD stage B and dogs without MMVD. In systemic hypertension, the concentration of NT-proBNP from the myocardium is thought to increase because systemic hypertension affects the left ventricle (LV). Therefore, we compared the serum NT-proBNP concentrations in dogs without MMVD, normotensive dogs with MMVD stage B, and systemic hypertensive dogs with MMVD stage B, to investigate the effect of systemic hypertension on NT-proBNP concentrations between dogs with MMVD stage B.

## Methods

### Study design and animals

Medical records were searched to identify dogs with stage B MMVD and those without MMVD that underwent cardiac examination between 2016 and 2021. Dogs were excluded if they had cardiac arrhythmia, pulmonary hypertension, left ventricular outflow tract obstruction, patent ductus arteriosus, or chronic kidney disease because these diseases can increase NT-proBNP concentrations. We measured blood pressure and conducted electrocardiography, radiography, echocardiography, hematology, blood chemistry, and NT-proBNP tests in dogs who visited regularly. The dogs included in this study had no underlying diseases (chronic kidney disease, hyperadrenocorticism, diabetes mellitus, pulmonary hypertension, and arrhythmia) affecting blood pressure. We grouped dogs without MMVD and normotensive or hypertensive dogs with MMVD stage B based on their history, physical examination, CBC, serum biochemistry, blood pressure, thoracic radiographs, abdominal ultrasound, electrocardiogram, and echocardiographic examination. Although hyperadrenocorticism was not diagnosed by the dexamethasone suppression test or ACTH stimulation test, there were no clinical symptoms and routine laboratory results related to hyperadrenocorticism such as polyuria, polydipsia, potbelly, or high ALP, and no stress pattern was observed on CBC. We excluded pulmonary hypertension by clinical symptoms and echocardiographic examination (echocardiographic signs of pulmonary hypertension: increased tricuspid regurgitation velocity, flattening of the interventricular septum, underfilling or decreased size of the left ventricle, pulmonary artery enlargement, right atrial enlargement, enlargement of the caudal vena cava), and arrhythmia by ECG examination.

We obtained informed consent from the owners of the dogs before performing diagnosis. This retrospective study was reviewed and approved by the Institutional Animal Care and Use Committee (KW-181210–1).

### MMVD B stage classification

**Fig. 1:**
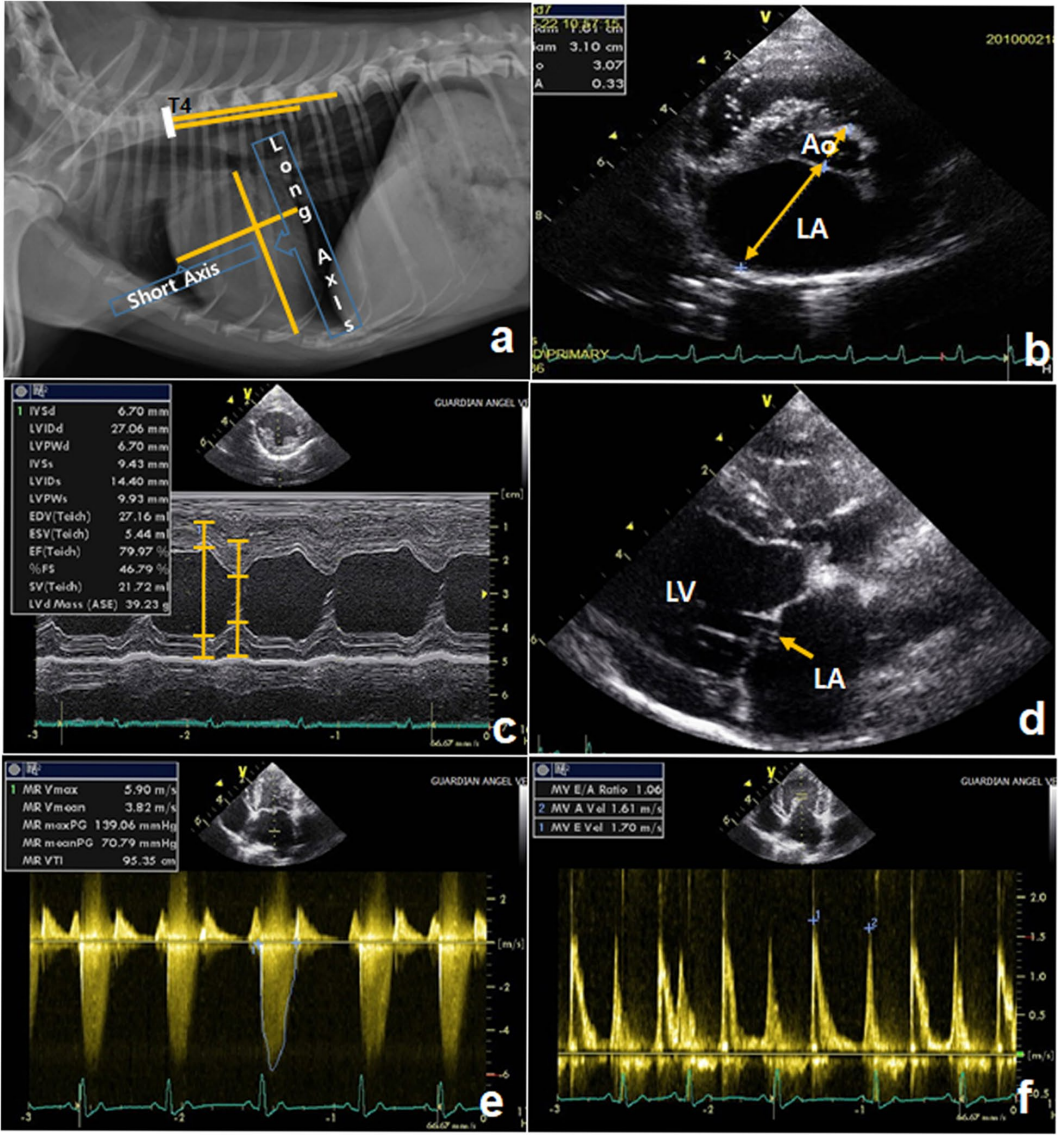
Thoracic radiographic and echocardiographic images of a dog with MMVD stage B. **a** Marked left atrial (LA) and left ventricular (LV) enlargements are observed. Measurement of the vertebral heart score (VHS). **b** The LA-to-aortic root ratio (LA/AO ratio: LA size divided by AO diameter) is measured at maximal LA size (end of ventricular systole) on a short-axis 2Dimage. **c** M-mode of the LV, measurement of LV internal dimension at end-diastole. **d** Mitral valve degeneration is seen in this 2D image. **e** Mitral valve regurgitation velocity (MR) on continuous-wave Doppler from a left long-axis 2D image. **f** E peak/A peak ratio on pulsed-wave Doppler from a left long-axis 2D image. LVIDd, Left ventricular internal diameter end diastole; E peak, peak mitral inflow velocity during early diastole; A peak, peak mitral inflow velocity at atrial contraction; LVPWd, Left ventricular posterior wall end-diastolic thickness

According to the ACVIM guidelines, MMVD is divided into stages A, B(B1, B2), C, and D [4]. In this study, we measured body weight, heart sound, heart rate, sleeping respiratory rate, rectal temperature, blood tests (ProCyteDx, IDEXX, Inc. Westbrook, ME, USA), and serum chemistry tests (NX500, FUJI, Tokyo, Japan) in dogs. We assessed clinical symptoms by monitoring respiration during rest and sleep, and by using auscultation and thoracic radiography. To evaluate cardiac functions, we performed radiography, electrocardiogram, and echocardiography [4]. Thoracic radiographs were acquired using the right lateral, ventrodorsal, left lateral, and dorsoventral views. We evaluated the outlines of the heart and lung space, and measured vertebral heart score (VHS) using thoracic radiographs [14] (Fig. 1a). The electrocardiogram (VET AT-1, SCHILLER, Switzerland) performed was used to check for arrhythmia based on conduction disturbance. The echocardiography used to examine the internal structure of the heart was non-invasively performed using an ultrasonic device (Vivid 7, GE, Milwaukee, WI, USA) with a 3.5- to 8.0-MHz phase-array probe. The LA/AO ratio was measured in the right parasternal short axis 2D image, and the LV internal diameter end diastole was measured in the right parasternal short axis M-mode. Mitral valve degeneration was diagnosed on the right parasternal long axis 2D image [15]. The MR velocity and the ratio of the E peak to A peak were measured on the left parasternal long axis 2D image (Fig. 1b, c, d, e, and f). After an overall cardiac evaluation, the dogs were classified as B stage [4, 16].

### Blood pressure measurement

According to the guidelines of ACIVM [5], blood pressure was mainly measured at the tail, and some individuals with short tails were measured at the legs. An indirect measurement method was used in small dogs because the Korotkoff sound was not detected in them as that in humans. The arterial blood flow sound was amplified using piezoelectric crystal detection (Doppler flow detector Model 811-B, Parks medical electronics, Inc. Aloha, Oregon USA) [17]. The size of the cuff was about 40% of the leg or tail circumference. The cuff height was maintained at the right atrial location during the measurement. The first measurement was discarded, and the average value of 5–7 measurements was used after re-measurement between 30 s and 1 min. We measured blood pressure before other examinations to reduce blood pressure changes [18]. The dogs were classified as having systemic hypertension when the measured systolic blood pressure was greater than 160 mmHg [5].

### Measurement of the blood concentration of NT-proBNP

All dogs were fasted for 12 h before blood collection. Blood samples were collected from the jugular or cephalic vein in K3-ethylenediaminetetraacetic acid tubes. The collected plasma samples were refrigerated and subjected to NT-proBNP measurement by IDEXX Laboratories, Inc., Seoul, South Korea. IDEXX Labs performed the NT-proBNP test using a commercial sandwich enzyme immunoassay.

### Statistical analysis

Data are presented as median [interquartile range] in the text and expressed using box-and-whisker plots in all the figures. A Shapiro–Wilk normalization test was performed together with nonparametric statistical analyses. Because the data did not follow a normal distribution, we used a nonparametric method. We compared the three groups using the Kruskal–Wallis test and Dunn’s test. Statistical significance was set at *p* < 0.05. We compared NT-proBNP concentrations between dogs without MMVD (*n* = 13), normotensive dogs with MMVD stage B (*n* = 30), and systemic hypertensive dogs with MMVD stage B (*n* = 7), and performed echocardiographic comparison among the three groups. We performed statistical analyses using IBM SPSS Statistics for Windows, Version 24.0 (IBM Corp., Armonk, NY, USA).

## Results

### Animals

The study population included 61 dogs that underwent cardiac examinations in 2016 and 2021 at a local hospital as part of a study evaluating NT-proBNP, blood pressure, and selected echocardiographic variables. In this study, we excluded dogs with arrhythmia (*n* = 3), pulmonary hypertension (*n* = 3), left ventricular outflow tract obstruction (*n* = 1), patent ductus arteriosus (*n* = 1), and chronic kidney disease (*n* = 3) because these diseases could elevate NT-proBNP concentrations. The study population included 30 normotensive dogs with MMVD stage B, 7 dogs with systemic hypertension with MMVD stage B, and 13 dogs without MMVD.

This study included 50 dogs that underwent NT-proBNP testing (13 dogs without MMVD, 30 normotensive dogs with MMVD stage B, and 7 systemic hypertensive dogs with MMVD stage B). The mean ages of the dogs were: 3 years (IQR, 2–12) for dogs without MMVD, 12 years (IQR, 10–13) for normotensive dogs with MMVD stage B, and 14 years (IQR, 12.5–14.5) for systemic hypertensive dogs with MMVD stage B. Dogs with MMVD, with or without systemic hypertension, were significantly older than those without MMVD (*p* = 0.005 among the groups). Among these dogs, 23 were males. Most of the dogs belonged to small breeds, with Maltese (*n* = 25) and Shih Tzu (*n* = 6) being the most dominant ones that accounted for 62% of the total 50 dogs included. The other breeds included Yorkshire Terrier (*n* = 1), Miniature Pinscher (*n* = 1), Pekingese (*n* = 1), Schnauzer (*n* = 1), Poodle (*n* = 1), Dachshund (*n* = 1), Mixed (*n* = 3), Wolf Dog (*n* = 1), Spitz (*n* = 1), Pompitz (*n* = 1), Bedlington Terrier (*n* = 1), Pomeranian (*n* = 3) and Cocker Spaniel (*n* = 3) (Table 1).

**Table 1:** Demographic distribution of the study population

**Variable**	Dogs without MMVD (Group 0, *n* = 13)	Normotensive dogs with MMVD stage B (Group 1, *n* = 30)	Systemic hypertensive dogs with MMVD stage B (Group 2, *n* = 7)
Age (years)			
Median (IQR)	3(2–12)	12(10–13)	14(12.5–14.5)
Sex			
Male	7	12	4
Female	6	18	3
BW (kg)			
Median (IQR)	4.24(3.2–5.9)	3.7(3.1–6.1)	6.5(2.8–11.8)
Blood pressure (mmHg)			
Median (IQR)	146(130–150)	142(130–147.5)	174(169–185)
Breed			
YT			1
MA	6	18	1
ST	2	4	
MP		1	
PE		1	
SC		1	
PO			1
DA		1	
MX		3	
WD	1		
SP	1		
POM		1	
BT			1
CS			3
PM	3		

Data obtained from the three groups were compared using the Kruskal–Wallis test (†) and Dunn’s test (*). †Significant differences (*p* < 0.05) according to the Kruskal–Wallis test. *:significant (*p* < 0.05) differences according to Dunn’s test are indicated by an asterisk. The ages of the three groups were compared: Group 0 vs. Group 1(*p* = 0.028*), Group 0 vs. Group 2 (*p* = 0.009*), Group 1 vs. Group 2 (*p* = 0.635), and Group 0 vs. Group 1vs. Group 2 (*p* = 0.005). Blood pressure was also compared between Group 0 and Group 1(*p* = 1*), Group 0 vs. Group 2 (*p* = 0.002*), Group 1 vs. Group 2 (*p* < 0.001*), and Group 0 vs. Group 1vs.Group 2 (*p* < 0.001†). *MMVD* myxomatous mitral valve disease, *IQR* Interquartile range, *BW* Body weight, *YT* Yorkshire Terrier, *MA* Maltese, *ST* Shih Tzu, *MP* Miniature Pinscher, *PE* Pekingese, *SC* Schnauzer, *PO* Poodle, *DA* Dachshund, *MX* Mixed, *WD* Wolf dog, *SP* Spitz, *POM* Pompitz, *BT* Bedlington terrier, *CS* Cocker spaniel, *PM* Pomeranian

### Serum biochemistry

Serum chemistry of 50 dogs confirmed no kidney disease, thyroid disease, hyperadrenocorticism, or diabetes (Table 2).

**Table 2:** Serum biochemistry values of normal and MMVD dogs. Data are presented as median (IQR)

**Variable**	Dogs without MMVD (Group 0, *n* = 13)	Normotensive dogs with MMVD stage B (Group 1, *n* = 30)	Systemic hypertensive dogs with MMVD stage B (Group 2, *n* = 7)	Reference Interval
Albumin (g/dL)	3.7(3.5–4.0)	3.5(3.3–3.7)	3.4(3.2–3.7)	2.6–4.0
Total Cholesterol (mg/dL)	234 (201.5–276.5)	214(173.0–268.5)	266.5 (245.3–314.8)	111–312
Bilirubin-Total (mg/dL)	0.1(0.1–0.2)	0.1(0.1–0.1)	0.1(0.1–0.1)	0.1–0.5
Calcium[Ca + +] (mg/dL)	11.3(10.8–12.5)	11.3(10.9–11.9)	10.6(10.0–11.5)	9.3–12.1
Protein-Total (g/dL)	7.1(7.0–7.3)	6.8 (6.5–7.2)	6.7(6.5–6.9)	5.0–7.2
ALT (U/L)	41.0(30.0–70.5)	50.0(40.0–75.8)	47.0(27.3–65.3)	17–78
Amylase (U/L)	585.0 (474.0–849.0)	578.0 (414.0–891.0)	536.0 (455.8–597.8)	200–1400
Glucose (mg/dL)	117.0 (107.0–126.5)	118.0 (107.5–125.5)	106.0 (102.8–110.3)	75–128
ALKP (U/L)	105.0 (58.5–298.5)	243.0 (160.3–351.8)	150.0 (113.5–202.0)	47–254
Phosphorus-Inorganic (mg/dL)	3.8(3.1–4.6)	3.7(3.1–4.2)	3.6 (3.2–3.8)	1.9–5.0
Creatinine (mg/dL)	0.9(0.7–1.15)	0.6(0.4–0.8)	0.7(0.5–0.8)	0.4–1.4
BUN (mg/dL)	25.9(19.6–29.0)	17.5(15.2–26.9)	19.9 (16.9–29.3)	9.2–29.2
Na + (mmol/L)	148.0 (146.3–150.5)	147.0 (145.0–149.0)	148.0 (146.5–148.0)	141–152
K + (mmol/L)	4.0(3.9–4.2)	4.3(4.2–4.7)	4.4 (4.2–4.6)	3.8–5.0
Cl- (mmol/L)	111.0 (108.8–114.0)	110.5 (106.3–112.0)	112.0 (111.0–116.0)	102–117
Globulin (g/dL)	3.3(3.2–3.5)	3.4(2.9–3.6)	3.2(3.0–3.4)	1.6–3.7

*MMVD* Myxomatous mitral valve disease, *IQR* Interquartile range, *ALT* Alanine aminotransferase, *ALKP* Alkaline phosphatase, *BUN* Blood urea nitrogen

### Comparison of the NT-proBNP levels among dogs without MMVD, normotensive dogs with MMVD stage B, and systemic hypertensive dogs with MMVD stage B groups

**Fig. 2:**
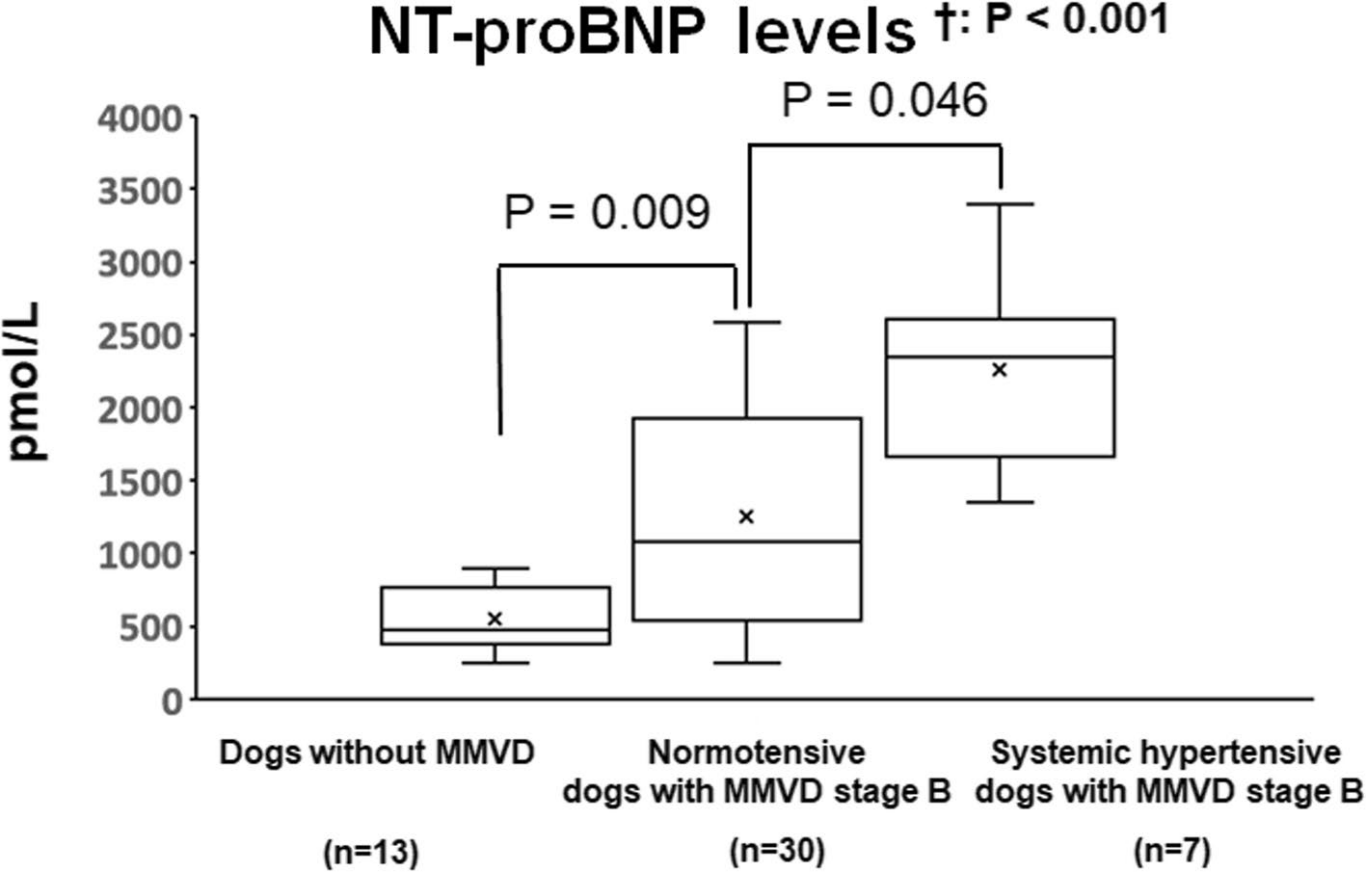
NT-proBNP levels in dogs without MMVD and normotensive and hypertensive dogs with MMVD stage B. Data are expressed using box-and-whisker plots. × indicates the mean. Data obtained from three groups are analyzed using the Kruskal–Wallis test (†) followed by Dunn’s test. NT-proBNP, N-terminal pro-B-type natriuretic peptide; MMVD, myxomatous mitral valve degeneration

Blood pressure was 146 (IQR, 130–150) mmHg in the group of dogs without MMVD, 142 (IQR, 130–147.5) mmHg in the group of normotensive dogs with MMVD stage B, and 174 (IQR, 169–185) mmHg in the group of dogs with systemic hypertension with MMVD stage B: Systemic hypertensive dogs with MMVD had significantly higher blood pressure than dogs without MMVD and normotensive dogs with MMVD (*p* < 0.001among groups).; the corresponding measured NT-proBNP concentrations were 504 (IQR, 430–774) pmol/L, 1083.5 (IQR, 574.8–1912.8) pmol/L, and 2345.0 (IQR, 1812.5–2533.0) pmol/L, respectively. A significant difference was observed between the three groups (Kruskal–Wallis test, *p* < 0.001). The NT-proBNP concentration was significantly higher in the group of normotensive dogs with MMVD stage B than in the group of dogs without MMVD (Dunn’s test, *p* = 0.009). Moreover, NT-proBNP concentration was significantly higher in the group of dogs with systemic hypertension with MMVD stage B than in the group of normotensive dogs with MMVD stage B (Dunn’s test, *p* = 0.046) (Fig. 2).

### Comparison of echocardiographic measurements among dogs without MMVD, normotensive dogs with MMVD stage B, and systemic hypertensive dogs with MMVD stage B groups

The LA/AO ratio, E peak, LVIDDN, and VHS were higher in the groups of normotensive dogs with MMVD stage B and dogs with systemic hypertension with MMVD stage B than in the group of dogs without MMVD. Mitral valve regurgitation velocity was significantly higher in the group of dogs with systemic hypertension with MMVD stage B 6.11 (IQR, 6.07–6.24) m/s than in the group of normotensive dogs with MMVD stage B 5.53 (IQR, 5.17–5.95) m/s (*p* = 0.006) (Table 3).

**Table 3:** Comparison of echocardiographic measurements among the three groups

Variable	Dogs without MMVD (Group 0, *n* = 13)	Normotensive dogs with MMVD stage B (Group 1, *n* = 30)	Systemic hypertensive dogs with MMVD stage B (Group 2,*n* = 7)	***p***–**value** (Kruskal–Wallis test)
Echo				
LA/AO				
Median (IQR)	1.47(1.25–1.51)	2.00 (1.87–2.26)	2.07 (1.81–2.34)	
***p***–**value** **(**Dunn’s test)	Group 0 vs. Group 1: < 0.001*			< 0.001†
	Group 0 vs. Group 2: 0.004*			
	Group 1 vs. Group 2: 1			
E peak(m/s)				
Median (IQR)	0.7(0.6–0.795)	1.21 (1.07–1.29)	1.15 (1.06–1.45)	
***p***–**value** **(**Dunn’s test)	Group 0 vs. Group 1: 0.001*			0.001†
	Group 0 vs. Group 2: 0.016*			
	Group 1 vs. Group 2: 1			
MR(m/s)				
Median (IQR)	ND	5.53 (5.17–5.95)	6.11 (6.07–6.24)	
***p***–**value** (Mann–Whitney U tests)	Group 1 vs. Group 2: 0.006^			–
LVIDDN				
Median (IQR)	1.3(1.24–1.345)	1.67 (1.63–1.90)	1.72 (1.57–1.77)	
***p***–**value** (Dunn’s test)	Group 0 vs. Group 1: < 0.001*			0.001†
	Group 0 vs. Group 2: 0.016*			
	Group 1 vs. Group 2: 1			
LVPWd/BW^0.232				
Median (IQR)	0.41(0.3–0.43)	0.43(0.38–0.50)	0.44(0.41–0.45)	0.430
VHS Median (IQR)	9.6(9.5–9.95)	10.60(10.03–10.80)	10.4(9.85–11.10)	
***p***–**value** **(**Dunn’s test)	Group 0 vs. Group 1: 0.041*			0.048†
	Group 0 vs. Group 2: 0.272			
	Group 1 vs. Group 2: 1			

Data obtained from the three groups were compared using the Kruskal–Wallis test (†) and Dunn’s test (*). †Significant differences (*p* < 0.05) according to the Kruskal–Wallis test. *:significant (*p* < 0.05) differences according to Dunn’s test are indicated by an asterisk. MR data did not follow a normal distribution, the Mann–Whitney U test (^) was used for comparisons between the two groups. ^^^Significant differences (*p* < 0.05) according to the Mann–Whitney U test. *MMVD* Myxomatous mitral valve degeneration, *IQR* Interquartile range, *LA/AO* Left atrial-to-aortic root ratio, *E peak* Peak mitral inflow velocity during early diastole, *MR* Mitral valve velocity, *ND* Not detectable, *LVIDDN* Left ventricular internal dimension in diastole normalized to body weight, *LVPWd* Left ventricular posterior wall end-diastolic thickness, *VHS* Vertebral heart score

## Discussion

In this study, NT-proBNP concentrations and echocardiographic parameters were measured and compared among normotensive and hypertensive dogs with MMVD stage B and dogs without MMVD to confirm the degree of stress on the ventricle. Echocardiographic variables included the LA/AO ratio, E peak, LVIDDN, LVPWd, VHS, and MR velocity. The NT-proBNP concentrations were significantly higher in dogs with MMVD stage B than in those without MMVD and were significantly higher in systemic hypertensive dogs with MMVD stage B than in their normotensive counterparts. The LA/AO ratio, E peak, and LVIDDN were significantly lower in the dogs without MMVD than in the dogs with MMVD stage B.

Mitral valve regurgitation is caused by the degeneration of the mitral valve. The amount of blood reflux increases as the degree of MR increases, resulting in LA enlargement as LA pressure increases [2, 15]. In this study, MR velocity was significantly higher in the systemic hypertension group than in the normotensive group (*p* = 0.006). The increase in NT-proBNP values in the systemic hypertensive dogs with MMVD stage B was due to the increase in the wall stress of the left ventricle as the MR velocity increased.

Hypertension can be subclassified as primary (essential) or secondary.

Approximately 90%–95% of cases in humans are primary, which is defined as a high blood pressure due to nonspecific lifestyle and genetic factors. The other 5%–10% of cases are categorized as secondary hypertension, which is defined as a high blood pressure due to an identifiable cause, such as chronic kidney disease, narrowing of the kidney arteries, or an endocrine disorder. Common diseases associated with hypertension in dogs include chronic kidney disease, hyperadrenocorticism, and diabetes mellitus. Some studies reported that 60%–90% of dogs with kidney failure develop hypertension. Hypertension also occurs in 70%–80% of dogs with hyperadrenocorticism, and 25%–45% of dogs with diabetes mellitus [5].

In this study, we excluded dogs with diseases known to be associated with secondary systemic hypertension. The included dogs with systemic hypertension and MMVD did not have clinical symptoms such as PUPD, potbelly, anorexia, or weight loss and had a normal range of routine laboratory parameters related to diseases that cause secondary hypertension. These parameters included ALP, GLU, BUN, CREA, CHOL, Na + , K + , Cl-, and no stress pattern on the CBC.

The most accurate method of measuring blood pressure is to measure the blood pressure directly by inserting an arterial cannula or pressure catheter. However, direct blood pressure measurements in small animals are generally difficult to perform because they require anesthesia. Therefore, indirect measurement using blood pressure measurement cuffs and blood pressure monitors is widely used for measuring blood pressure in small animals. In humans, blood flow is blocked using a pressure gauge and cuff, and the Korotkoff sound is sensed using a stethoscope while gradually releasing the air. However, because small animals do not have a Korotkoff sound as in humans, arterial blood flow is generally measured indirectly by amplification using piezoelectric crystal detection (i.e., a Doppler probe) [18]. Thus, in this experiment, we measured blood pressure using Doppler ultrasonography. Other indirect measurement methods include oscillometry and high definition oscillometry. In this study, blood pressure was measured according to the ACVIM guidelines [5]. When the measured systolic or diastolic blood pressure exceeds 160 mmHg or 95 mmHg, respectively, it is defined as systemic hypertension [5]. In small animals, systolic blood pressure is important because it is generally associated with the risk of end-organ damage, and additional treatment is needed if animals are confirmed as having hypertension.

In this study, systemic hypertension was confirmed by repeatedly measuring elevated blood pressure, and dogs that were not hypertensive due to stress were selected. Among these dogs, we included dogs with primary hypertension after confirming that they did not have secondary hypertension through clinical symptoms and blood tests. The underlying cause of primary hypertension is unclear; however, in humans, it is reportedly linked to increased cardiac output (the amount of blood pumped out every time the heart contracts) or an increase in peripheral vascular resistance.

Heart diseases increase the blood levels of vasoactive substances because of various neuroendocrine responses. Measurement of the circulating concentrations of these substances (cardiac biomarkers) can provide useful clinical information. Although many biomarkers for heart diseases have been identified, BNP and NT-proBNP are the most clinically useful in human and veterinary medicine [6, 7]. This was used to adjunctly predict the stage of heart disease [19, 20]. Natriuretic peptides are stored in granules bound to the membranes of the atria and ventricles. They are released into the blood as a reaction to myocardial wall deformation. Protein degradation of the precursor molecule proBNP results in a biologically active C-terminal BNP, a 32-amino acid peptide, and a deactivated 76-amino acid N-terminal fragment (NT-proBNP). In dogs, the blood half-life of C-terminal BNP is approximately 1.57 min [19] and the NT-proBNP half-life is approximately 1–2 h which makes it more suitable for use as a biomarker because of its gradual reduction. In humans, BNP and NT-proBNP are used as reliable biomarkers in heart disease or heart failure. A study on humans investigated the utility of NT-proBNP in the emergency assessment of acute heart failure or dyspnea, and the effect of hypertension on the heart [8–11]. Another study reported that NT-proBNP levels were eightfold higher in patients with hypertension than in healthy controls, and that NT-proBNP was a strong prognostic factor in the diagnosis and prognosis of heart failure [20]. In particular, patients with a history of cardiovascular disease have a high NT-proBNP concentration and show a sevenfold increase in the incidence of cardiovascular disease than patients with a low NT-proBNP concentration and no cardiovascular disease [20]. In veterinary clinics, NT-proBNP is used as adjunct evidence to differentiate heart disease from other causes of dyspnea in dogs and cats [21, 22]. A correlation exists between the severity of hypertrophic cardiomyopathy and the serum concentration of natriuretic peptide in cats [23]. It is used to adjunct diagnose the relationship between the heart and dyspnea, the heart and respiratory disease, and the heart and pleural effusion, as well as systemic hypertension [24]. The NT-proBNP is used in dogs as supplement for diagnosing mitral valve disease, arrhythmia, myocardial disease, pulmonary hypertension, heart disease, and chronic valvular heart disease (CVHD) [21, 24, 25]. A recent study suggested that NT-proBNP concentrations are elevated when cats are hypertensive and that NT-proBNP can be used adjunctly to predict hypertension treatment outcomes [13]. To the best of our knowledge, no previous study has compared NT-proBNP concentrations among normotensive and hypertensive dogs with MMVD stage B and dogs without MMVD. This study confirmed that hypertensive dogs with MMVD stage B had significantly higher concentrations of NT-proBNP than normotensive dogs with MMVD stage B.

These results suggest that systemic hypertension may increase NT-proBNP. Therefore, when NT-proBNP testing is performed in a dog with heart disease, blood pressure measurement must be included and the clinical symptoms, cardiac test results, and NT-proBNP concentrations should be evaluated comprehensively.

The limitation of this study was the low number of dogs with MMVD stage B and systemic hypertension. Future studies should be carried out to obtain more data on dogs with MMVD stage B and systemic hypertension as well as to compare NT-proBNP concentrations between normotensive and hypertensive dogs with MMVD stage B. A dog with systemic hypertension in MMVD stage B has increased NT-proBNP; therefore, it would be meaningful to check how fast the period of progression to heart failure is, in such a dog, than in a normotensive dog with MMVD stage B. In reality, long-term monitoring studies are unlikely to proceed because treatment is performed in dogs with systemic hypertension. Therefore, a follow-up study investigating the change in NT-proBNP concentration following hypertension therapy may have significant clinical implications.

## Conclusions

Our study demonstrated that systemic hypertension increases NT-proBNP by increasing the burden on the left ventricle. Stress on the left ventricle is greater and NT-proBNP concentrations are higher in systemic hypertensive dogs with MMVD stage B than in normotensive dogs with MMVD stage B. Therefore, clinicians should be aware that NT-proBNPcould be elevated in the presence of systemic hypertension.

## Data Availability

All datasets are available in the main manuscript.

## Abbreviations

Ao: Aorta
A peak: Peak mitral inflow velocity at atrial contraction
CVHD: Chronic valvular heart disease
CW: Continuous-wave
E peak: Peak mitral inflow velocity during early diastole
IQR: Interquartile range
LA: Left atrium
LA/AO: Left atrium to aortic root diameter ratio
LV: Left ventricle
LVIdDN: Left ventricular end diastolic diameter normalized for body weight
NT-proBNP: N-Terminal Pro-B-Type Natriuretic Peptide
MMVD: Myxomatous mitral valve degeneration
MR: Mitral valve regurgitation
PW: Pulse wave
VHS: Vertebral heart score
